# Anti-Atherosclerotic Action of Agmatine in ApoE-Knockout Mice

**DOI:** 10.3390/ijms18081706

**Published:** 2017-08-04

**Authors:** Anna Wiśniewska, Rafał Olszanecki, Justyna Totoń-Żurańska, Katarzyna Kuś, Aneta Stachowicz, Maciej Suski, Anna Gębska, Mariusz Gajda, Jacek Jawień, Ryszard Korbut

**Affiliations:** 1 Department of Pharmacology, Jagiellonian University Medical College, 31-531 Krakow, Poland; anna.niepsuj@interia.pl (A.W.); jzuranska@gmail.com (J.T.-Ż.) katarzyna_poskrubek@o2.pl (K.K.); stachowicz.aneta@gmail.com (A.S.); macieksuski@gmail.com (M.S.); mfgebska@cyf-kr.edu.pl (A.G.); mmjawien@cyf-kr.edu.pl (J.J.); mfkorbut@cyf-kr.edu.pl (R.K.); 2 Department of Histology, Jagiellonian University Medical College, 31-034 Krakow, Poland; mmgajda@cyf-kr.edu.pl

**Keywords:** atherosclerosis, agmatine, apoE-knockout mice, fatty liver metabolism

## Abstract

Atherosclerosis is an inflammatory disease in which dysfunction of mitochondria play an important role, and disorders of lipid management intensify this process. Agmatine, an endogenous polyamine formed by decarboxylation of arginine, exerts a protective effect on mitochondria and modulates fatty acid metabolism. We investigated the effect of exogenous agmatine on the development of atherosclerosis and changes in lipid profile in apolipoprotein E knockout (apoE-/-) mice. Agmatine caused an approximate 40% decrease of atherosclerotic lesions, as estimated by en face and cross-section methods with an influence on macrophage but not on smooth muscle content in the plaques. Agmatine treatment did not changed gelatinase activity within the plaque area. What is more, the action of agmatine was associated with an increase in the number of high density lipoproteins (HDL) in blood. Real-Time PCR analysis showed that agmatine modulates liver mRNA levels of many factors involved in oxidation of fatty acid and cholesterol biosynthesis. Two-dimensional electrophoresis coupled with mass spectrometry identified 27 differentially expressed mitochondrial proteins upon agmatine treatment in the liver of apoE-/- mice, mostly proteins related to metabolism and apoptosis. In conclusion, prolonged administration of agmatine inhibits atherosclerosis in apoE-/- mice; however, the exact mechanisms linking observed changes and elevations of HDL plasma require further investigation.

## 1. Introduction

Despite remarkable progress in medicine, cardiovascular diseases, among them atherosclerosis and atherosclerosis-related organ injury, are still one of the major causes of morbidity [1]. Atherosclerosis is a chronic inflammatory disease of the arterial wall, characterized by endothelial dysfunction, infiltration of inflammatory cells, and gradual formation of atherosclerotic plaque with its lipid-rich core and rupture-prone fibrous cap [2]. In every stage of atherogenesis, disorders of lipid metabolism (hypercholesterolemia, elevated levels of free fatty acids in the blood) and disturbances of lipoprotein turnover (elevated level of low density lipoproteins (LDL) and lowered level of high density lipoproteins (HDL)) intensify this process.

Mitochondria are organelles that accomplish several vital functions, including oxidative phosphorylation and metabolism of fatty acids, as well as integration of signaling for apoptosis and cellular senescence. Many factors (e.g., angiotensin II, tumor necrosis factor alpha (TNF-α)) that play the main role in pathogenesis of atherosclerosis have been shown to cause mitochondrial damage. Indeed, mitochondrial injury, associated with derangement of their metabolic and signaling capabilities and the increased production of reactive oxygen species (ROS), has been described in various cells within the vessel wall (especially endothelial cells) [3,4]. Either disturbances in lipid/lipoprotein metabolism or the development of non-alcoholic fatty liver disease (NAFLD) has been recently connected to impairment of mitochondrial function in the hepatocytes [5]. The protection of mitochondria may represent a new promising strategy to attenuate atherosclerosis [6].

Agmatine is an endogenous polyamine that is synthesized by decarboxylation of L-arginine by arginine decarboxylase (ADC). It is present in plasma and can be selectively concentrated in several organs (i.e., in the liver) [7,8,9]. Agmatine has been shown to exert a wide array of biologic effects as follows: it regulates blood pressure, inhibits the proliferation of smooth muscle cells, and exhibits anti-inflammatory and neuroprotective function [10,11,12]. Agmatine also exhibits a protective effect on mitochondria, where it is actively concentrated by an energy-dependent mechanism [13,14,15,16]. The multifaceted metabolic and molecular effects of long-term administration of agmatine have been comprehensively studied [17]. Agmatine, among others, has been shown to directly stimulate mitochondrial oxidation of fatty acids in the liver [18]. Recently, exogenous agmatine has been found to inhibit atherogenesis in cholesterol-fed rabbits and this action was attributed to its antioxidant and anti-inflammatory potential (i.e., inhibitory action on inducible nitric oxide synthase (iNOS)). In cholesterol-fed rabbits agmatine was able to decrease plasma levels of LDL and increase the levels of HDL [19]. However, the profound metabolic and inflammatory effects related to massive feeding with cholesterol make this model unreliable for detailed studies of mechanisms by which agmatine affects lipid metabolism [20]. The widely accepted model devoid of such drawback is apoE-knockout (apoE-/-) mice, which spontaneously develop hypercholesterolemia, dyslipidemia, and arterial lesions on the chow diet [21]. The current study was designed to investigate the influence of prolonged treatment with agmatine on the development of atherosclerosis and changes in lipid profile in apoE-/- mice using morphological, biochemical, and molecular methods. We hypothesized that in the apoE-knockout mice the liver may represent an important target organ, and the hepatocyte mitochondria may represent the target organelle for exogenous agmatine. As mitochondrial proteomics represents a valuable tool for the study of metabolic disorders and the mechanisms of actions of metabolic drugs [22], we also applied the methods of differential proteomics to elucidate the effect of exogenous agmatine on the liver mitochondria of apoE-/- mice.

## 2. Results

### 2.1. Body Weight

The mean body weight in the control (30.69 ± 2.37 g) and agmatine-treated (32.06 ± 2.79 g) groups did not differ (*p* = 0.14). Also food intake was similar in both groups. 

### 2.2. Effects of Agmatine on Atherosclerosis

**Figure 1:**
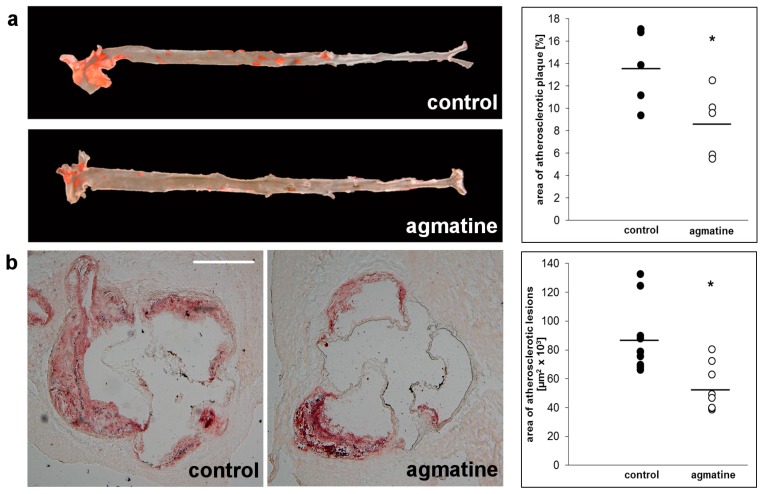
Oil Red O stained representative aortas in control group and agmatine-treated group. Percentage of area occupied by atherosclerotic lesions measured by the en face method in the control group and agmatine-treated group (**a**); representative micrographs showing Oil Red O stained lesions in control group and agmatine-treated group. Atherosclerotic lesions area is measured by the cross-section method in control group and agmatine-treated group (**b**). Black lines indicate mean values (* *p* < 0.05). Each dot represents a single mouse in control (black dots, *n* = 10) or agmatine-treated (white dots, *n* = 8) group. The scale bar represents 500 μm.

Significant reduction of atherosclerotic lesions resulted in the aortas of apoE-knockout mice when treated with agmatine. Measured by en face method, the percentage of area occupied by atherosclerosis lesions in the agmatine-treated group was 8.72 ± 3.1%, whereas the percentage in the control group was 13.7 ± 3.6% (*p* < 0.05) (Figure 1a). Nearly 40% of the difference in surface of atherosclerotic lesions was revealed with the cross-section method of aortic roots. The areas of Oil Red O stained changes were 52,786 ± 16,499 μm^2^ in the agmatine-treated group versus 87,582 ± 23,847 μm^2^ in the control group (Figure 1b).

**Figure 2:**
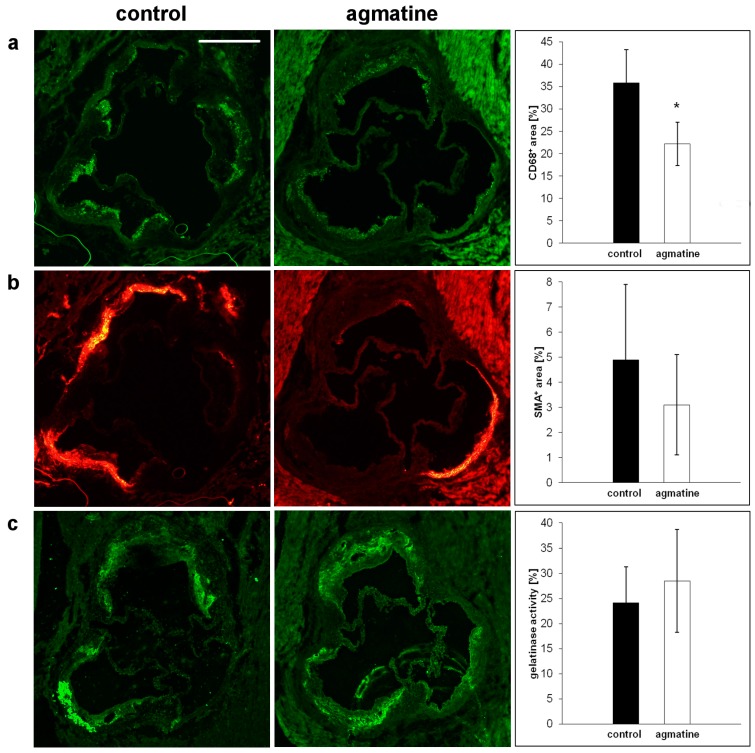
Representative micrographs showing immunohistochemical staining of aortic roots from control and agmatine-treated apoE-knockout mice. The figure shows immunohistochemical visualization and quantitative analysis of CD68-positive macrophages (green) (**a**), smooth muscle α-actin (SMA)*-*positive cells (red) (**b**), and gelatinase activity (green) (**c**). (* *p* < 0.05; Mean ± SD). The scale bar represents 500 μm.

Immunohistochemical staining showed that not only the size of the atherosclerotic plaque but also the composition of the lesions was favorably changed by agmatine treatment. The plaque area covered by CD68 immunopositive macrophages was reduced in agmatine-treated mice compared to the control group (22.2 ± 4.8% vs. 35.8 ± 7.5%, *p* < 0.05) (Figure 2a), while the content of smooth muscle cells in the fibromuscular cap was similar in both groups (4.9 ± 3.0% vs. 3.1 ± 2.0%, *p* = 0.16) (Figure 2b). Agmatine treatment did not significantly change gelatinase activity within the plaque area detected by in situ zymography (28.51 ± 10.23% vs. 24.17 ± 7.1%) (*p* = 0.24) (Figure 2c).

Treatment with agmatine significantly influenced serum lipid profiles in apoE-/- mice. The level of HDLs was significantly higher in the agmatine-treated group compared to the control animals, while there were no differences in the levels of LDLs or triglycerides. Agmatine showed the tendency to increase total cholesterol levels, although the changes did not reach statistical significance (Table 1). Plasma levels of inflammatory markers monocyte chemoattractant protein 1 (MCP-1) and interleukin-12 (IL-12) in the agmatine group showed a slight but significant increase compared to the control group (Table 2).

### 2.3. RT^2^ Profiler PCR Arrays

**Figure 3:**
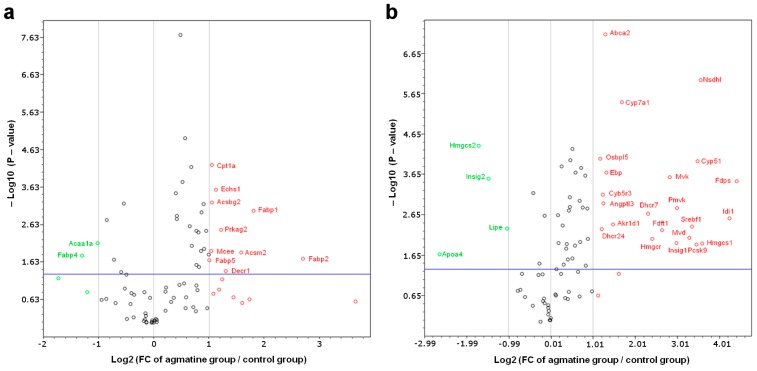
Volcano plot graphs of mouse fatty acid metabolism PCR array (**a**) and mouse lipoprotein signaling and cholesterol metabolism PCR array (**b**). Graphs show the log_2_ of fold change of gene expression between the agmatine group and control group versus *p* value from the t test. ° up-regulation, ° down-regulation. Blue line indicates threshold 0.05 for *p* value.

Using Profiler PCR Arrays liver gene expression was analyzed. The expression of 11 out of the 84 fatty acid metabolism genes was significantly changed in the agmatine-treated group compared to the control group; among them nine genes were significantly upregulated (Table 3, Figure 3a). Also, as a result of agmatine treatment, the expression of 26 genes related to lipoprotein signaling and cholesterol biosynthesis was found to be changed, whereof 22 of were significantly upregulated (Table 4, Figure 3b).

### 2.4. Influence of Agmatine on Liver Mitoproteome

**Figure 4:**
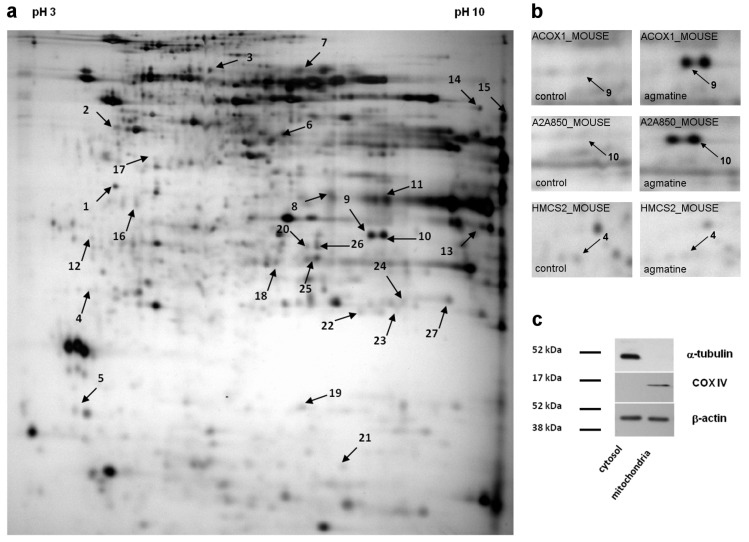
Representative 2D map of mitochondrial proteins expressed in apoE-knockout mice (**a**), with magnifications of spot pairs corresponding to peroxisomal acyl-Coenzyme A oxidase 1 (ACOX1), acyl-Coenzyme A oxidase 1, palmitoyl (A2A850), and hydroxymethylglutaryl-CoA synthase (HMCS2) (**b**). The arrows mark 27 spots showing differences between control and agmatine-treated group (spot numbers correspond to the numbers in Table 5). Purity of mitochondrial fraction was assessed by the Western blotting method, showing the absence of cytosolic a-tubulin l in mitochondrial fraction (**c**).

**Figure 5:**
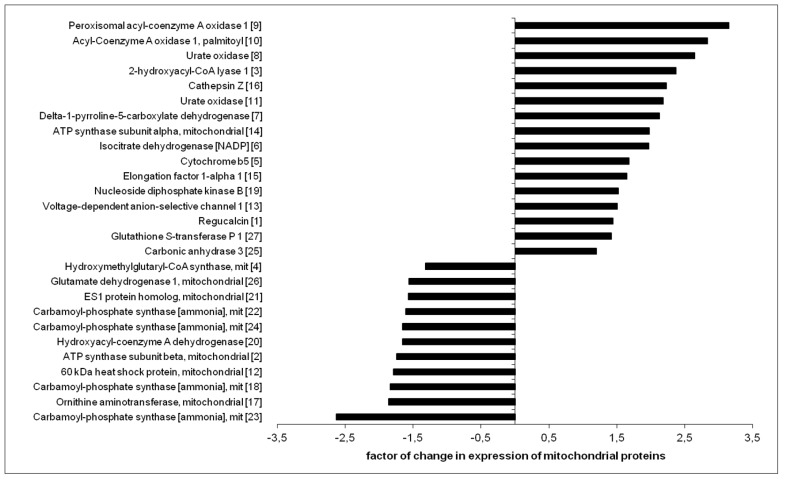
Relative changes in expression of mitochondrial proteins in agmatine-treated apoE-/- mice, compared to control apoE-/- mitochondria. Corresponding spot numbers are shown in brackets (*n* = 3 per group).

The representative 2DE gel image of liver mitochondrial proteins of apoE-knockout, as well as selected pairs of spots showing differences between apoE-knockout and agmatine-treated apoE-knockout mice, is presented in Figure 4a,b, respectively. Table 5 lists the LC-MS/MS analysis from numbered spots showing significant differences. Collectively, 27 differentially expressed spots were detected and identified by software (PDQuestTM) and analyzed by mass spectrometry. Figure 5 shows the quantitative results of differences in expression of mitochondrial proteins. The accuracy of the isolation protocol and the purity of the mitochondrial fractions were assessed by immunoblotting for α-tubulin and cytochrome c oxidase (COX-IV) (Figure 4c).

## 3. Discussion

### 3.1. Effect of Agmatine on the Development and Structure of Atherosclerotic Lesions in ApoE-Knockout Mice

Our study indicates that exogenous agmatine, used for four months, significantly inhibits the development of atherosclerosis in apoE-knockout mice. So far, the anti-atherogenic action of exogenous agmatine has been reported only once in cholesterol-fed rabbits [19]. The model of apoE-/- mice, which develop dyslipidemia and atherosclerotic plaques spontaneously, is devoid of overwhelming metabolic and inflammatory disturbances caused by cholesterol overloading, and is more reliable for studies of mechanisms of the anti-atherosclerotic action of potential drugs [21]. The daily dose in our experiments (20 mg/kg/day) should be considered as a medium one as the oral agmatine dose practiced in literature in mice and rats ranges from 1 to 200 mg/kg. Importantly, our administration of agmatine did not lead to abnormalities in food intake and weight gain in mice as well as it did not cause any observable adverse effects. Thus, in our experiment exogenous agmatine appeared to be an effective and safe inhibitor of atherogenesis in apoE-knockout mice.

In our study, agmatine not only reduced the area of atherosclerotic lesions but also changed their structure by reducing the number of macrophages in atherosclerotic plaques. The action of agmatine could be connected to the stabilization of atherosclerotic plaques as macrophages can play a key role in atherosclerotic plaque destabilization and rupture. However, the model of atherosclerosis used in this study does not allow for a direct assessment of risk of atherosclerotic plaque destabilization (the plaque rupture in apoE-knockout mice fed with a standard diet is very rare), so verification of the hypothesis about the plaque-stabilizing effect of agmatine requires further research.

Despite the inhibitory effect on macrophages accumulation in plaques, agmatine did not reduced the level of proinflammatory proteins in the blood. In contrast, it showed a tendency to increase plasma levels of MCP-1 and IL-12. In the context of the general inhibitory effect of agmatine on the development of atherosclerosis, such a surprising effect requires future verification, perhaps by in vitro studies using endothelial cells (major source of MCP-1) and phagocytic/dendritic cells (major source of IL-12).

### 3.2. Mechanisms of Anti-Atherosclerotic Action of Agmatine in ApoE-Knockout Mice

An approximate threefold increase of the level of HDL in the blood was the most striking action of agmatine observed in apoE-knockout mice. At the same time, agmatine did not significantly change the level of other lipoproteins. Numerous studies, both retrospective and long-term prospective follow-up, have shown that a low HDL level is associated with an increased risk of coronary events [23] and high HDL levels have a protective effect in this regard [24,25]. The beneficial effect of HDL is primarily associated with reverse cholesterol transport (RCT), a pathway that transports cholesterol from extrahepatic cells and tissues to the liver and intestine for excretion, by reducing accumulation of cholesterol in the wall of the arteries. In this context, HDLs are capable not only of counteracting the development of atherosclerosis but may also reverse the current changes in the arteries [26]. In our study, agmatine strongly elevated the HDL level in the blood of apoE-/- mice, which gives rise to the hypothesis that such an action might be responsible for its anti-atherosclerotic effect. The question arises: what could be the molecular mechanism(s) of influence of agmatine on the lipid and lipoprotein metabolism in apoE-knockout mice? There could be many potential pathways targeted by agmatine in this regard, for example the increase in the blood HDL level could depend either on the increased formation of young HDL or on the inhibition of the metabolism of mature lipoproteins or the degradation of their major apolipoprotein—apoA I. Considering the complexity of liver lipid and lipoprotein metabolism, and the wide array of biological and metabolic activities of agmatine, we have assessed its influence on mRNA expression of factors related to the hepatic metabolism of fatty acids, cholesterol, and lipoproteins; we have also undertaken open comprehensive analysis of agmatine-derived changes in the expression of proteins in mitochondrial fraction isolated from the liver of apoE-/- mice.

### 3.3. The Effect of Agmatine on mRNA Expression of Factors Involved in Hepatic Lipid Metabolism

The PCR arrays showed a significant increase of expression of factors involved in fatty acid metabolism in agmatine-treated group mice as compared to the control group. Agmatine increased the expression of two genes encoding the enzyme acyl-CoA synthetase (*Acsbg2* and *Acsm2*), which belongs to a large family of enzymes catalyzing the first step in the metabolism of fatty acids. *Acsbg2* (acyl-CoA synthetase bubblegum family member 2) encodes an enzyme that mediates activation of long-chain fatty acids for both synthesis of cellular lipids, and their degradation via beta-oxidation; *Acsm2* (acyl-CoA synthetase medium-chain family member 2) activates medium-chain length fatty acids. Moreover, agmatine has increased the expression of mRNA for *Cpt1* (carnitine palmitoyltransferase I), a key enzyme involved in the transport of fatty acids into mitochondria and *Decr1* (2,4-dienoyl CoA reductase 1, mitochondrial), an enzyme that participates in the further steps of beta oxidation (mainly unsaturated fatty acids) [27]. In our experiment, agmatine also increased the expression of mRNA for *Mcee* (methylmalonyl CoA epimerase), an enzyme that catalyzes the conversion of D- and L-methylmalonyl-CoA during the degradation of branched chain amino acids, odd chain-length fatty acids, or cholesterol [28].

The expression of genes for fatty-acid-binding protein (FABP) was altered with agmatine treatment. Agmatine increased the expression of the gene for L-FABP (liver-type fatty acid binding protein), but decreased the expression of the gene for A-FABP (adipocyte-type fatty acid binding protein). A-FABP, in addition to facilitating the fatty acid metabolism, may be involved in inflammatory processes, as was shown, so that its expression in macrophages is induced by oxidized low density lipoprotein (oxLDL) and proinflammatory cytokines [29,30]. It has also been shown that A-FABP inhibitors protected against atherosclerosis in apoE-knockout mice [31,32]. Our data demonstrate the broad effect of agmatine on the expression of genes coding factors involved in fatty acid metabolism: enzymes involved in liver β-oxidation, the transportation of fatty acids into mitochondria, and proteins involved in the intracellular circulation of lipids. Our results are consistent with functional ones reported by Nissim et al., according to which agmatine increases β-oxidation of fatty acids in isolated mitochondria and perfused rat liver [18]. However, whether or not an increase of β-oxidation in the liver may link agmatine with increased HDL levels requires further investigation. Niacin, a model compound raising the blood level of HDL, is also known to increase β-oxidation of fatty acids in the liver [13,33,34]. In addition to the aforementioned effect on the expression of enzymes involved in mitochondrial fatty acids metabolism, agmatine has reduced the expression of the gene encoding of the enzyme involved in β-oxidation in the peroxisomes—*Acaa1a* (acetyl-Coenzyme A acyltransferase 1A). Therefore, the evaluation of the effect of agmatine on the activity of individual enzymes involved in fatty acids metabolism requires further research.

What is more, in our experiment agmatine changed the expression of mRNAs for a number of factors related to the cholesterol metabolism in the liver of apoE-/- mice. Observed changes are not easy to interpret. They could indicate the intensification of hepatic cholesterol synthesis by agmatine; however, many of aforementioned factors show various activities. For example, *Dhcr24* also codes seladin 1, which is a multifunctional protein regulating cholesterol metabolism and building lipid membrane rafts, and also shows antioxidant and anti-apoptotic properties [35]. Upon treatment with agmatine two isoforms of one protein may behave differently (e.g., for *Insig1*, the expression increased, while for *Insig2*, the expression decreased), while both perform similar function. Therefore, full explanation of the effect of agmatine on hepatic pathways of cholesterol metabolism as well as the role of this effect in the anti-atherosclerotic effect of agmatine certainly require further investigation.

### 3.4. Effect of Agmatine on Liver Mitoproteome

Agmatine has been shown to accumulate in mitochondria [13]. Mitochondria are also organelles at the crossroad of the pathways of fatty acid oxidation and the selected pathways of cholesterol and lipoprotein metabolism. Thus, in order to evaluate the changes in expression of proteins in mitochondria isolated from the liver of control and agmatine-treated apoE-/- mice, we used methods of differential proteomics in our study. Several identified proteins differing in expression between agmatine-treated and control mice appeared to be enzymes involved in the oxidation of fatty acids. Agmatine has an increased expression of ACOX1 (peroxisomal acyl-coenzyme A oxidase 1), which catalyzes the bottleneck reaction in beta-oxidation of very long-chain fatty acids in peroxisomes [36]. Importantly, it can also residue in the mitochondria and the deficiency in ACOX (isoform 1 and 2) has been shown to contribute to non-alcoholic fatty liver disease, both in mice and in humans [37]. Another protein with increased expression in the agmatine-treated group was HACL1 (2-hydroxyacyl-CoA lyase 1). This enzyme has two important roles in alpha oxidation: the degradation of phytanic acid, and shortening of 2-hydroxy long-chain fatty acids so that they can enter beta oxidation [38].

The agmatine-elicited changes in the mitochondrial expression of enzymes involved in the metabolism of fatty acids are not straightforward; for example, the expression of HCDH (hydroxyacyl-coenzyme A dehydrogenase), an enzyme involved in fatty acid oxidation, has been reduced in the agmatine-treated group. Agmatine also decreased the expression of HMCS (hydroxymethylglutaryl-CoA synthase), an enzyme that catalyzes the reaction in which acetyl-CoA condenses with acetoacetyl-CoA to form 3-hydroxy-3-methylglutaryl-CoA (HMG-CoA). The widely known cytosolic form of HMCS that is, HMCS 1 takes part in one of the first stages of cholesterol synthesis, while the mitochondrial form HMCS2 participates in the formation of ketones. Clearly the effects of agmatine on mitochondrial proteins involved in lipid metabolism require further research.

A number of mitochondrial proteins not related directly to metabolism with expressions targeted by agmatine were revealed by proteomic approach. Some of them can be classified as proteins that modulate oxidative stress, apoptosis, and inflammatory responses. Agmatine has increased the expression of anti-apoptotic GST P1 (glutatione S-transferase P1) that has been reported to suppress c-Jun N-terminal kinase (JNK)-related apoptosis [39,40,41,42]. In turn, agmatine increased the expression of cathepsin Z (cathepsin, Z/X/P), a cysteine-type lysosomal protease, whose deficiency leads to the accelerated aging of cells [43]. In this context, the action of agmatine on GST P1 and cathepsin Z can be targeted to counteract apoptosis and the aging of liver cells. It is difficult to unambiguously interpret the inhibitory effect of agmatine on the expression of Hsp60 (heat shock protein 60). The overexpression of Hsp60 in cardiomyocytes protects against apoptosis [44]. On the other hand, Hsp60 is involved in the maturation of the main apoptotic executioner, caspase 3 [45]. Similarly, the biological meaning of agmatine-derived changes in the mitochondrial levels of several other proteins with a complex function is not easy to interpret: P5CD (P5CDH, P5CS, delta-1-pyrroline-5-carboxylate dehydrogenase), the enzyme that plays an important role in the metabolism of proline, arginine, and glutamate; NDPK (nucleoside diphosphate kinase), a transcription factor and an enzyme involved in the metabolism of nucleotides [46]; CAIII (carbonic anhydrase III), the enzyme that can protect cells from oxidative stress and participate in the metabolism of fatty acids [47]; or the regucalcin calcium binding protein, playing a large role in maintaining intracellular calcium homeostasis [48]. Undoubtedly, the analysis of the effects of agmatine on the expression of proteins involved in defense against ROS, regulation of apoptosis and inflammatory processes requires careful functional studies.

### 3.5. Conclusion and Future Directions

We have found that prolonged administration of agmatine significantly inhibited atherosclerosis in apoE-/- mice; such an action was associated with the elevation of HDL in plasma. The comprehensive analysis of its influence on mRNA expression of factors related to the hepatic metabolism of fatty acids, cholesterol, and lipoproteins, as well as agmatine-derived changes in the expression of proteins in liver mitochondria, revealed many traces of potential relevance: agmatine appeared to increase liver expression of enzymes related to fatty acid oxidation, and changed levels of proteins that can modulate oxidative stress, apoptosis, and inflammatory responses. 

Yet, the exact mechanisms linking observed changes with HDL rise and anti-atherosclerotic action require further clarification. The future studies should focus on the possible influence of agmatine on HDL function and the formation/degradation of main HDL-related proteins in the liver (i.e., apoA I). Also, the analysis of the direct cellular action of agmatine on the vessel wall (endothelial and smooth muscle cells, macrophages and foam cells) would shed new light on its anti-atherosclerotic action. Noteworthy, the stimulation of lipolysis by agmatine outside the liver, i.e., in thermogenic fat tissue and skeletal muscles, could also be the case, as such action has been reported to elevate plasma HDL levels [49]. Finally, an important question arises as to whether the anti-atherogenic action of agmatine involves imidazoline receptors or occurs in a non-receptor manner. Intriguingly, the activation of imidazoline I-1 receptors has been linked to the hypolipemic effect [50]. Each of these questions needs to be resolved in future mechanistic studies.

## 4. Materials and Methods 

### 4.1. Animal Experiments

Male apoE-knockout mice on the C57BL/6J background were obtained from Taconic (Ejby, Denmark). The mice were housed in air-conditioned rooms (22.5 ± 0.5 °C, 50 ± 5% humidity) with 12-h dark/12-h light cycles, with unconstrained access to food and water, in the Animal House of Chair of Immunology of JUMC. Eight week-old mice were put on a chow diet made by Wytwórnia Pasz Morawski (Kcynia, Poland). Animals were randomly divided into two groups: the control group (apoE-knockout mice w/o treatment, on chow diet as above, *n* = 10) and agmatine-treated mice (*n* = 8). In this group, agmatine (Sigma-Aldrich, St. Louis, MO, USA) was mixed without heating with the same diet and administered to mice at a dose of 20 mg/kg of body weight per day (according to calculation taking into account average body mass of mouse and its daily diet requirement). After four months on the experimental diet the mice were injected with 1000 IU of fraxiparine (Sanofi-Synthelabo, France) into the peritoneum, then were killed using a carbon dioxide chamber. Next, the blood was collected and hearts, aortas, and livers were dissected. All animal procedures were approved by the Jagiellonian University Ethical Committee on Animal Experiments (No. 74/2011, 8 June 2011).

### 4.2. Analysis of Atherosclerotic Plaque

The aortas were prepared en face and stained Oil Red O (Sigma-Aldrich, St. Louis, MO, USA). The aortic lesion area and total aortic area were calculated using LSM Image Browser software.

The hearts with the ascending aorta were embedded in OCT compound (CellPath, Newtown, UK), snap frozen and sectioned (10 μm thickness) for histological and immunohistochemical analysis, according to the standardized cross-section protocol, as described before [51,52]. To evaluate the lesion area and plaque collagen content, nine sections per animal were stained Oil Red O (Sigma-Aldrich, St. Louis, MO, USA). Immunohistochemistry was performed with antibodies against CD68 (dilution 1:800; Serotec, Kidlington, UK) and smooth muscle α-actin (SMA) (dilution 1:800; Sigma-Aldrich, St. Louis, MO, USA). In situ zymography was performed to demonstrate non-specific activity of gelatinases using the standard protocol [53]. All section images were captured using an Olympus Camedia DP71 digital camera and analyzed using LSM Image Browser software (Zeiss, Jena, Germany).

### 4.3. Biochemical Methods

The blood was collected from the right ventricle and centrifuged for 10 minutes, 1000 *g* at 4 °C. Plasma was harvested and stored in −80 °C until assayed. The level of total cholesterol, triglycerides, and low and high density lipoproteins (LDL and HDL) were measured using an enzymatic method on a Cobas 8000 analyzer (Roche Diagnostics, Indianapolis, IN, USA). In addition, levels of some inflammation markers, such as interleukin 12 (IL-12), vascular cell adhesion protein 1 (VCAM-1), monocyte chemoattractant protein 1 (MCP-1), and serum amyloid A (SAA) were measured by ELISA using commercially available kits (R&D Systems, Minneapolis, MN, USA).

### 4.4. RT^2^ Profiler PCR Arrays

Total RNA was isolated from the liver tissues using QIAzol Lysis Reagent (QIAGEN, Valencia, CA, USA), using a standard protocol. The concentration of RNA was determined by measuring the absorbance in an EPOCH Microplate Spectrophotometer (BioTek Instruments Inc., Winooski, VT, USA). The A260/A280 ratio was greater than 1.9 in all samples. The same amount of total RNA (1 μg) for each sample was reverse-transcribed into cDNA using the RT^2^ First Strand Kit (SABiosciences, Frederick, MD, USA), according to the manufacturer’s instructions. The RT^2^ Profiler PCR Arrays were used to analyze the expression levels of 84 key genes involved in fatty acid metabolism (PAMM-007Z, SABiosciences, Frederick, MD, USA), or lipoprotein signaling and cholesterol metabolism (PAMM-080Z, SABiosciences, Frederick, MD, USA). PCR reactions were performed using the 7900HT Fast Real-Time PCR System (Applied Biosystems, Foster City, CA, USA), and the data was analyzed using the web-based program of RT^2^ Profiler PCR Array Data Analysis. A 2-fod cut off threshold was used to define up or down regulation of the genes analyzed.

### 4.5. Two-Dimensional Electrophoresis (2-DE) and Gel Image Analysis

The isolation of the mitochondria fraction from the freshly-harvested livers was performed at 4 °C, as previously described [51]. Mitochondrial pellets were resuspended in 0.5 mL of lysis buffer (9.5 M urea, 4% CHAPS, 2% DTT, 0.5% Bio-Lyte 3–10 (Bio-Rad, Hercules, CA, USA), a mix of protease inhibitors (Sigma-Aldrich, St. Louis, MO, USA)). Then, samples were vortexed, incubated at 25 °C for 30 min, and centrifuged at 12,000× *g* for 15 min. The protein concentration was determined in the harvested supernatant with the Bradford method [54]. Next, samples were purified with 2-DE Clean-up kit (GE Healthcare, Wilmington, MA, USA), resuspended in 300 μL of rehydration buffer (8 M urea, 0.5% CHAPS, 0.2% DTT and 0.2% Bio-Lyte 3–10), and loaded on linear 3–10 immobilized pH gradient 17 cm strips (Bio-Rad, Hercules, CA, USA). The strips were focused with a multistep voltage gradient from 400 to 3500 V (max 50 mA/IPG strip, 20 °C) for a total of 66 kVh. Then, the strips were reduced and alkylated in buffer (6 M urea, 30% glycerol, 2% SDS and 0.01% bromophenol blue) with the addition of 1% *w*/*v* DTT (20 min) and 4.8% *w*/*v* iodoacetamide (20 min). Second dimension (SDS-PAGE) polyacrylamide gels (12% T, 2.6% C) were performed using the Protean II xi system (Bio-Rad, Hercules, CA, USA). After electrophoresis the gels were fixed in ethanol:acetic acid:water solution (4:1:5 *v*/*v*/*v*) and visualized by silver staining using the Plus One silver staining kit (GE Healthcare, Wilmington, MA, USA) with modifications to provide subsequent mass spectrometry analysis [55]. Silver-stained gel images were taken using a GelDoc XR scanner (Bio-Rad, Hercules, CA, USA) and analyzed with PDQuest™ 8.0.1 (Bio-Rad, Hercules, CA, USA) software dedicated do gel image analysis, quantification, and statistical validation. In total, three biological replicates per group, each in two technical replicates (*n* = 6 gel images per group) were analyzed. A student’s *t*-test, implemented in PDQuest™, was used to reveal statistically significant differences (*p* < 0.05) in the protein expression, which were further analyzed with LC MS/MS system to identify proteins of interest.

### 4.6. LC MS/MS

Protein spots of interest were excised from the gel and then destained, reduced, alkylated, and digested with modified trypsin (Sigma-Aldrich, St. Louis, MO, USA), according to the protocol described by Shevchenko et al. [56]. Peptide mixtures were resuspended in 0.1% TFA and injected in an Acclaim PepMap100 RP C18 75 μm i.d.× 25 cm column (LC Packings/Dionex) via a trap column (PepMap100 RP C18 300 μm i.d. (inner diameter) × 5 mm column, LC Packings/ Dionex). The peptides were separated in 90 min 0–40% B phase linear gradient (buffer A: 5% acetonitrile, 0.1% formic acid; buffer B: 95% acetonitrile, 0.1% formic acid) by a Switchos/UltiMate 3000 RSLC nano HPLC system (LC Packings/Dionex, USA) with a flow rate of 300 nL/min and applied on-line to a LCQ (Thermo Finnigan, San Jose, CA, USA) ion-trap mass spectrometer. The ESI ion source parameters were: ion spray voltage 1.5 kV, capillary temperature 200 °C, and capillary voltage 10 V. The full scan mode (270–1600 Da), followed by three MS/MS scans of the most intense ions, were used to collect the spectra. Data analysis was performed by the X!Tandem search algorithm (the GPM Organization) and Trans-Proteomic Pipeline (TPP) software (Institute for Systems Biology) with the following parameters: enzyme: trypsin, taxonomy: mouse (SwissProt), missed cleavage sites allowed: 2, variable modification: oxidation of methionine, fixed modification: carbamidomethyl, selected device and parent δm: ion trap (4 Da), and peptide fragment mass tolerance: 0.4 Da.

### 4.7. Immunoblotting

Immunoblotting of cytochrome c oxidase (COX-IV) and α-tubulin was used to assess the purity of mitochondrial fractions, which was described previously [51]. The specific primary antibodies were as follows: 1:5000 ANTI-COX-IV (Abcam, Cambridge, MA, USA), 1:250 ANTI-alpha-tubulin (Sigma-Aldrich, St. Louis, MO, USA). Bands images were taken using an ImageQuant Las 500 (GE Healthcare, Chalfont, UK).

### 4.8. Statistical Analysis

The results are expressed as mean ± SD. The nonparametric Mann-Whitney U test (en face, Oil Red O and IHC data) or *t*-test (other methods) were used for statistical analysis of the data. *p* < 0.05 is considered to be statistically significant.

## Figures and Tables

**Table 1:** Plasma lipids level in control and agmatine-treated groups, presented as mean ± SD.

	TC (mmol/L)	TG (mmol/L)	HDL (mmol/L)	LDL (mmol/L)
control	13.8 ± 0.3	1.4 ± 0.2	3.7 ± 0.9	8.2 ± 0.8
agmatine	19 ± 4.1 (*p* = 0.052)	1.7 ± 0.6 (NS)	8.6 ± 1.8 *	11.7 ± 2.4 (NS)

TC, total cholesterol; TG, triglyceride; HDL, high density lipoproteins; LDL, low density lipoproteins; NS, non-significant difference between group; * *p* < 0.05.

**Table 2:** Vascular Cell Adhesion Protein 1 (VCAM-1), Interleukin-12 (IL-12), Monocyte Chemoattractant Protein 1 (MCP-1), and Serum Amyloid A (SAA) in a control and agmatine-treated groups, presented as mean ± SD.

	VCAM-1 (ng/mL)	IL-12 (pg/mL)	MCP-1 (pg/mL)	SAA (ng/mL)
control	9.09 ± 0.34	8.95 ± 0.52	45.16 ± 6.96	84,222 ± 20,109
agmatine	10.11 ± 1.16 (NS)	10.57 ± 0.82 *	64.82 ± 5.65 *	101,360 ± 54,611 (NS)

NS, non-significant difference between group; * *p* < 0.05.

**Table 3:** The genes involved in fatty acid metabolism that showed differential expression between agmatine group vs. control group (fold change > 2.0, *p* < 0.05).

Gene Name	Description	Fold Change	*p* Value (*t*-Test)
*Acsbg2*	Acyl-CoA synthetase bubblegum family member 2	2.08	0.000608
*Acsm2*	Acyl-CoA synthetase medium-chain family member 2	2.99	0.013220
*Cpt1a*	Carnitine palmitoyltransferase 1a, liver	2.07	0.000061
*Decr1*	2,4-dienoyl CoA reductase 1, mitochondrial	2.47	0.041195
*Fabp1*	Fatty acid binding protein 1, liver	3.51	0.001004
*Fabp2*	Fatty acid binding protein 2, intestinal	6.53	0.019459
*Fabp5*	Fatty acid binding protein 5, epidermal	2.01	0.021016
*Mcee*	Methylmalonyl CoA epimerase	2.06	0.011877
*Prkag2*	Protein kinase, AMP-activated, gamma 2 non-catalytic subunit	2.33	0.003263
*Acaa1a*	Acetyl-Coenzyme A acyltransferase 1A	−2.04	0.007525
*Fabp4*	Fatty acid binding protein 4, adipocyte	−2.47	0.015985

**Table 4:** The genes involved in lipoprotein signaling and cholesterol biosynthesis that showed differential expression between agmatine group vs. control group (fold change > 2.0, *p* < 0.05).

Gene Name	Description	Fold Change	*p* Value (*t*-Test)
*Abca2*	ATP-binding cassette, sub-family A (ABC1), member 2	2.46	0
*Akr1d1*	Aldo-keto reductase family 1, member D1	2.79	0.003913
*Angptl3*	Angiopoietin-like 3	2.37	0.001165
*Cyb5r3*	Cytochrome b5 reductase 3	2.36	0.000713
*Cyp51*	Cytochrome P450, family 51	11.18	0.000105
*Cyp7a1*	Cytochrome P450, family 7, subfamily a, polypeptide 1	3.22	0.000004
*Dhcr24*	24-dehydrocholesterol reductase	2.31	0.005083
*Dhcr7*	7-dehydrocholesterol reductase	4.96	0.002081
*Ebp*	Phenylalkylamine Ca2+ antagonist (emopamil) binding protein	2.49	0.000205
*Fdft1*	Farnesyl diphosphate farnesyl transferase 1	6.22	0.005329
*Fdps*	Farnesyl diphosphate synthetase	21.31	0.000338
*Hmgcr*	3-hydroxy-3-methylglutaryl-Coenzyme A reductase	5.33	0.008786
*Hmgcs1*	3-hydroxy-3-methylglutaryl-Coenzyme A synthase 1	12.10	0.011450
*Idi1*	Isopentenyl-diphosphate delta isomerase	18.94	0.002732
*Insig1*	Insulin induced gene 1	7.87	0.011090
*Mvd*	Mevalonate (diphospho) decarboxylase	9.78	0.008416
*Mvk*	Mevalonate kinase	7.08	0.000262
*Nsdhl*	NAD(P) dependent steroid dehydrogenase-like	11.77	0.000001
*Osbpl5*	Oxysterol binding protein-like 5	2.25	0.000091
*Pcsk9*	Proprotein convertase subtilisin/kexin type 9	10.98	0.012371
*Pmvk*	Phosphomevalonate kinase	7.94	0.001513
*Srebf1*	Sterol regulatory element binding transcription factor 1	10.17	0.004453
*Apoa4*	Apolipoprotein A-IV	−6.23	0.021159
*Hmgcs2*	3-hydroxy-3-methylglutaryl-Coenzyme A synthase 2	−3.28	0.000045
*Insig2*	Insulin induced gene 2	−2.78	0.000292
*Lipe*	Lipase, hormone sensitive	−2.05	0.004930

**Table 5:** Differentially expressed proteins in liver mitochondria of agmatine-treated vs. control apoE-knockout mice *.

No.	Protein	SwissProt Accession Number	Molecular Mass (kDa)	pI	Unique Peptides	Total Peptides	Protein Coverage (%)	Fold Change
1	Regucalcin	RGN_MOUSE	33.4	4.94	7	10	27	1.440
2	ATP synthase subunit beta, mitochondrial	ATPB_MOUSE	56.3	5.19	5	8	11	−1.740
3	2-hydroxyacyl-CoA lyase 1	HACL1_MOUSE	63.6	5.89	7	12	14	2.370
4	Hydroxymethylglutaryl-CoA synthase, mit	HMCS2_MOUSE	56.8	8.65	2	4	4.1	−1.320
5	Cytochrome b5	CYB5_MOUSE	15.2	4.96	2	4	16.,4	1.680
6	Isocitrate dehydrogenase [NADP]	IDHC_MOUSE	46.6	6.48	5	8	13	1.970
7	Delta-1-pyrroline-5-carboxylate dehydrogenase	AL4A1_MOUSE	61.8	8.58	2	2	2.70	2.130
8	Urate oxidase	URIC_MOUSE	35	8.48	5	6	17	2.650
9	Peroxisomal acyl-Coenzyme A oxidase 1	ACOX1_MOUSE	74.6	8.64	5	6	9.40	3.150
10	Acyl-Coenzyme A oxidase 1, palmitoyl	A2A850_MOUSE	74.6	8.64	6	15	11	2.840
11	Urate oxidase	URIC_MOUSE	35	8.48	11	19	33	2.180
12	60 kDa heat shock protein, mitochondrial	CH60_MOUSE	60.9	5.91	3	5	5.1	−1.790
13	Voltage-dependent anion-selective channel 1	VDAC1_MOUSE	32.3	8.55	5	9	20	1.510
14	ATP synthase subunit alpha, mitochondrial	ATPA_MOUSE	59.7	9.22	4	8	7.10	1.980
15	Elongation factor 1-alpha 1	EF1A1_MOUSE	50.1	9.1	4	4	8.00	1.650
16	Cathepsin Z	CATZ_MOUSE	34	6.13	4	13	17.6	2.230
17	Ornithine aminotransferase, mitochondrial	OAT_MOUSE	48.3	6,19	4	8	9.8	−1.860
18	Carbamoyl-phosphate synthase [ammonia], mit	CPSM_MOUSE	164.5	6.48	4	7	3.9	−1.840
19	Nucleoside diphosphate kinase B	NDKB_MOUSE	17.3	6.97	7	13	48.,60	1.520
20	Hydroxyacyl-coenzyme A dehydrogenase	HCDH_MOUSE	34.4	8.76	2	5	6.10	−1.660
21	ES1 protein homolog, mitochondrial	ES1_MOUSE	28.1	9	2	2	6.80	−1.570
22	Carbamoyl-phosphate synthase [ammonia], mit	CPSM_MOUSE	164.5	6.48	3	4	2.40	−1.610
23	Carbamoyl-phosphate synthase [ammonia], mit	CPSM_MOUSE	164.5	6.48	4	6	3.50	−2.630
24	Carbamoyl-phosphate synthase [ammonia], mit	CPSM_MOUSE	164.5	6.48	5	11	5.30	−1.660
25	Carbonic anhydrase 3	CAH3_MOUSE	29.3	6.97	4	6	15	1.200
26	Glutamate dehydrogenase 1, mitochondrial	DHE3_MOUSE	61.3	6.71	4	5	7.90	−1.560
27	Glutathione S-transferase P 1	GSTP1_MOUSE	23.6	8.13	3	6	20	1.420

pI indicates isoelectric point; * *p* < 0.05; *n* = 3 per group.
